# Seasonal Effects on Gene Expression

**DOI:** 10.1371/journal.pone.0126995

**Published:** 2015-05-29

**Authors:** Anita Goldinger, Konstantin Shakhbazov, Anjali K. Henders, Allan F. McRae, Grant W. Montgomery, Joseph E. Powell

**Affiliations:** 1 University of Queensland Diamantina Institute, The Translational Research Institute, Brisbane, Queensland 4102, Australia; 2 The Queensland Brain Institute, The University of Queensland, Brisbane, Queensland 4072, Australia; 3 Queensland Institute of Medical Research, Herston, Brisbane, QLD 4006, Australia; University of North Carolina at Charlotte, UNITED STATES

## Abstract

Many health conditions, ranging from psychiatric disorders to cardiovascular disease, display notable seasonal variation in severity and onset. In order to understand the molecular processes underlying this phenomenon, we have examined seasonal variation in the transcriptome of 606 healthy individuals. We show that 74 transcripts associated with a 12-month seasonal cycle were enriched for processes involved in DNA repair and binding. An additional 94 transcripts demonstrated significant seasonal variability that was largely influenced by blood cell count levels. These transcripts were enriched for immune function, protein production, and specific cellular markers for lymphocytes. Accordingly, cell counts for erythrocytes, platelets, neutrophils, monocytes, and CD19 cells demonstrated significant association with a 12-month seasonal cycle. These results demonstrate that seasonal variation is an important environmental regulator of gene expression and blood cell composition. Notable changes in leukocyte counts and genes involved in immune function indicate that immune cell physiology varies throughout the year in healthy individuals.

## Introduction

The variation of RNA transcription levels within a population (P), is driven by both genetic (G) and environmental (E) factors (Eq (1)):

$$\begin{array}{c} \sigma_{P}^{2}=\sigma_{G}^{2}+\sigma_{E}^{2} \end{array}$$ (1)

Gene expression studies in humans have aimed to quantify the total contribution of genetic variation underlying RNA level variation within a population and to identify genetic loci that contribute to that variation [1] [2]. The proportion of phenotypic variance explained by genetic variance is termed heritability (*H* ^2^) and can be estimated using information from related [3] and unrelated [4] [5] individuals. The RNA levels of transcripts measured using high-throughput arrays have moderate to high heritability estimates, with 42% of transcripts having additive genetic variance *h* ^2^ > 0.3 and 5% with *h*2 > 0.5 [6] [7]. Expression quantitative trait loci (eQTL) mapping studies have been extremely successful in identifying many loci that contribute to the heritable variation [8] [9] [10] [11]. However, environmental variation, which can be considered as 1- *H* ^2^, makes the largest contribution to variation in RNA expression levels [12]. Identifying and quantifying the influence of the environmental factors can provide a more thorough understanding of the differences in expression levels between individuals and between populations. Knowledge of environmental effects will also provide information on gene function and potentially gene-environment interactions, which will aid in understanding how expression profiles are affected by certain environmental conditions, such as geographical location [13].

One environmental factor that has a well-documented effect is seasonal variation. Changes in gene regulation have been associated with seasonal effects such as photoperiod in animals [14] [15] and plants [16] [17]. Previous research into the effect of seasonal variation in humans has focused only on a subset of genes [18] or cluster of genes [19] [20] and failed to identify many genes whose expression levels change in response to the season.

Seasonal changes between months of the year include but are not limited to differences in day length [21], ultraviolet (UV) index [22], humidity [23] and temperature [24], all of which could potentially influence expression levels either independently or interactively. Several health conditions are affected by seasonal changes, including asthma [25], cardiovascular disease [26], depression [27], diabetes [28], bipolar disorder [29], schizophrenia [30], migraine [31] and multiple sclerosis [32] [33]. Environmental changes between seasons also influence infection rates of influenza and respiratory syncytial virus [34] and vitamin D deficiency has been attributed to seasonal UV changes [35] [36]. Identifying genes, the expression levels of which change in response to the season could potentially shed light on some of the mechanisms that might be driving these health conditions. Here we report results from a systematic, genome-wide analysis of the effect of season on gene expression levels in a human population. We identified significant blood cell count changes in erythrocytes, leukocytes and platelets associated with seasonality and enrichment for expressed cellular gene markers for lymphocytes. Furthermore, after correcting for blood cell counts, we identified 135 probes whose expression levels were significantly associated with 12-month seasonal cycle.

## Materials and Methods

### Ethics Statement

This study used previously published data, deposited in GEO under accession number GSE33321. The research and study design were approved by the University of Queensland Human Ethics Review Board and the QIMR Berghofer Medical Research Institute Institutional Review Board for Research on Human Subjects. All participants gave informed written consent.

### Samples

This study comprised of 606 individuals from 246 families in the Brisbane System Genetics Study (BSGS) [37]. Genotype, gene expression and cell counts were measured for each individual.

### Genotyping

All individuals were genotyped on an Illumina 610-Quad Beadchip (Illumina Inc, San Diego, CA) by the Scientific Services Division at deCODE Genetics, Iceland. Full details of the genotyping procedure are given in [37].

### Gene expression

RNA levels were measured from whole blood collected with a PAXgeneTM tube (QIAGEN, Valencia, CA). The expression levels were quantified on an Illumina HumanHT-12 v4.0 Beadchip. Samples were randomized across the chip to minimize batch effects due to family, sex and age. Full details of the procedures used to generate the expression levels are given in [37]. Pre-processing of the microarray data, including calculation of average bead signal, removal of outliers and the calculation of detection p-values, was performed in the Illumina software Genome studio. Probes were considered significantly expressed above background noise at a *p* < 0.05. All probes falling below this threshold were considered non-expressed and denoted as such for further analysis. Probes that did not map to characterized Ref-Seq genes were removed. Probes with non-expression in > 50 of samples were excluded, leaving 13,311 probes for further analysis.

### Cell counts

Cell counts measured in BSGS include individual measures of single cell types, along with measures representing a composite of multiple cell types. For example, total white blood count includes measures of several cell types such as monocytes, lymphocytes, basophils, neutrophils and eosinophils. We chose to correct for the individual blood cell types, rather than composite measures. The cell types that were selected for correction were red blood (RBC), platelets (PLT), monocytes (MONO), basophils (BASO), neutrophils (NEUT), eosinophils (EOS), B-cells (CD19), Two subtypes of T-cells (CD4, CD8) and NK cells (CD56). Cell counts were log transformed and converted to z-scores. Linear regression was used to correct expression levels for effects due to cellular composition.

### Normalization

A rank-based inverse normal transformation (INT) was used to transform probe expression to a normal distribution. The normalization was done using the R package GenABEL [38]. As the BSGS contains related individuals, the polygenetic (cryptic and family) effects were removed by fitting the relationship matrix (***A***), determined using an identity-by-state (IBS) Genomic Relationship Matrix (GRM) in software package, Genome-wide Complex Trait Analysis (GCTA) [39]:

$$\begin{array}{c} y_{1}=g+e_{1} \end{array}$$ (2)

Where $g\sim N(0,\text{A}\sigma_{g}^{2})$ and $e_{1}\sim N(0,\text{I}\sigma_{e_{1}}^{2})$. Variation in expression levels can be attributed to batch effects such as chip and chip processing. Corrections were made for batch effects using Eq (3).

$$\begin{array}{c} y_{2}=X\beta +\mathrm{Zb}+e_{2} \end{array}$$ (3)

Where ***y*_2_ = *e*_1_**, ***Z*** is the incidence matrix for the chip ID fitted as a random effect (***b***) with $\text{b}\sim N(0,\text{I}\sigma_{e_{2}}^{2})$. Fixed effect covariates (***X***) were selected from a list of recorded batch effects using forward step-wise regression with model selection based on the lowest Akaike information criterion (AIC). Covariates that demonstrated significant association with gene expression levels included chip position, age, sex and length of sample storage, which is the difference between the date that the tissue sample was collected and the date that the mRNA was extracted. The values calculated from ***e*_2_** (Eq (3)) were used for further analysis and referred to as the “uncorrected” dataset.

### Cell count correction

The expression dataset was corrected for cell count using Eq (4):

$$\begin{array}{c} y_{3}=X\beta +e_{3} \end{array}$$ (4)

Where $y_{3}\;=\;e_{2},\;e_{3}\sim N(0,I\sigma_{e_{3}}^{2})$ and ***X*** is the fixed effect cell count covariates selected previously. The values obtained in ***e*_3_** (Eq (4)) were used for further analysis and referred to as the “corrected” dataset.

### Conversion to time series

BSGS tissue samples were collected over a six-year period, between February 2005 and March 2010. Expression levels and cell counts were averaged by the month of sample collection, creating a monthly time series for each probe. Of the 606 samples, 597 were collected between February 2005 and February 2008. The remaining 9 samples, which were collected between March 2008 and March 2010, were excluded from further time series analysis due to the low number of samples per month.

### Seasonal decomposition

The gene expression and cell count time series data were decomposed into seasonal (s), trend (t) and error components (*ϵ*) using loess function [40].

$$\begin{array}{c} y_{4}=g\left(t\right)+g\left(s\right)+e_{4} \end{array}$$ (5)

Where *y*_4_ are the residuals from ***e*_2_** from Eq (2) for the uncorrected analysis or ***e*_3_**, from Eq (3) for the corrected anlaysis. *g*(*s*) and *g*(*t*) are estimated by loess smoothing functions, which allow the estimation of repeating periodic variation without any constraint to a particular cyclical pattern. The trend component represents the overall changes that occur over the whole time series.

### Autocorrelation

Autocorrelation within time series data indicates the presence of periodic repeating patterns. A Ljung-Box test [41] was used to test for significant levels of autocorrelation in the *g*(*s*) estimates:

$$\begin{array}{c} Q=n\left(n+2\right)\sum_{k=1}^{h}\frac{{\hat{r}}_{k}^{2}}{n-k} \end{array}$$ (6)

Where *n* is the sample size, *k* is the lag, ${\hat{r}}_{k}$ is the autocorrelation, and *h* is the number of lags [41]. The test statistic (*Q*) follows a chi-square distribution with *h* degrees of freedom.

### Cosinor regression

Cyclic seasonal patterns, which have periodical cycles repeating over set time frames, can be modelled by the cosine function:

$$\begin{array}{c} f\left(t\right)=a\times cos[\left(\frac{2\pi t}{T}\right)-\theta ] \end{array}$$ (7)

Where *t* = month (1–12 for January to December), *T* = time period (in months) over which the cycle repeats, *a* = amplitude and *θ* = horizontal shift or phase of the cosine function [42]. This transformation creates the cosinor regression model [43]:

$$\begin{array}{c} y_{4}=\beta_{0}+\beta_{1}\times sin\left(\frac{2\pi t}{T}\right)+\beta_{2}\times cos\left(\frac{2\pi t}{T}\right)+e_{4} \end{array}$$ (8)

Where *y*_4_ = *s*, the seasonal component from Eq (5). Cosine and sine curves with repeating cycles (*T*) of 12 months were fitted. Significance was determined with ANOVA F-statistic and multiple testing accounted for using Bonferroni correction.

### Measured weather variables

The cosinor model was applied to 12 measured environmental variables collected for each month: mean maximum temperature, mean minimum temperature, mean daily ground minimum temperature, mean rainfall, mean number of rainy days, maximum wind gust speed, mean daily sunshine, mean daily solar exposure (MJ/m^2^)), mean number of sunny days, mean number of cloudy days, and mean daily evaporation (all obtained from the Australian Bureau of Meteorology—http://www.bom.gov.au/) and mean UV level (obtained from the Australian Radiation Protection and Nuclear Safety Agency—http://www.arpansa.gov.au/). The weather values for each individual used in this study can be found in S1 Table. The association of weather variables to a 12-month repeating cosine curve was determined with a Kendall tau rank correlation test. Kendall tau rank correlation is a non-parametric test that determines dependence between two ordinal variables.

### Biological enrichment analysis

Probes that demonstrated significant association to cyclic seasonal variation were tested for biological enrichment using DAVID (v6.7) [44] [45]. Functional annotation clustering was used to identify groups with shared annotation. Statistical significance of the clusters is given by an enrichment scores, where a score > = 1.3 is equivalent to a p-value of 0.05. Molecular pathways were identified from the Kyoto Encyclopaedia of Genes and Genomes (KEGG) pathway enrichment implemented by DAVID functional annotation tool. Significance of Gene Ontology (GO)-terms obtained from KEGG pathway enrichment and the functional annotation chart was determined using modified the Fisher’s exact test [44] [45] and corrected for multiple testing using the Benjamini-Hochberg false discovery rate (FDR) [46].

### Blood cell specific markers

Enrichment for blood cell-specific markers was determined using the userListEnrichment() function in the WGCNA R package [47]. This function compares the gene list obtained from the seasonal analysis to 11 published gene lists that are representative markers for blood cells including red blood cells, lymphocytes, leukocytes and platelets. The function tests for significant overlap between each list of genes and the cell markers using a hypergeometric test. Significant enrichment was determined by a study-wide significance threshold of *p* < 0.05/11 [48].

## Results

### Decomposition of time series data

**Fig 1:**
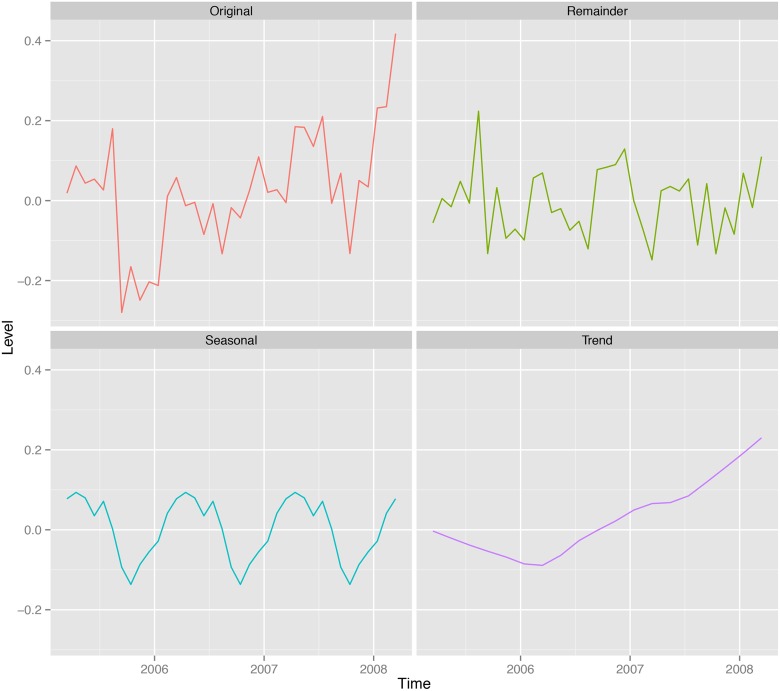
Time series decomposition for TRIM23 (ILMN_1752741) using loess decomposition. Original = The raw time-series data for the probe. Seasonal = The regular cyclic component. Trend = The linear drift over time. Remainder = The irregular (error) component that is not explained by the seasonal and trend components.

The Brisbane Systems Genetics Study (BSGS) dataset [37], comprising gene expression levels for 606 individuals and 13,311 probes, were decomposed into seasonal, trend and irregular (remainder) components using the loess smoothing function (see Fig 1 and Methods). This enables regular cyclic components for each probe to be isolated from residual or background noise.

### Effect of season on gene expression

Cosinor regression was used to test for effect of season (based on when the expression levels were sampled) for each of the time series adjusted probes. Cosinor regression is a linear model with sine and cosine terms that estimate the parameters of repeating cyclic variation across multiple phases (see Methods). To investigate the effect of season on blood cell counts, we performed the cosinor regression analysis on expression levels that had been adjusted for cell counts (“corrected”, see Methods) and unadjusted (“uncorrected”).

Significant associations with season at study-wide threshold of p < 0.05/13,311 were identified for 169 (uncorrected) and 135 (corrected) probes (Table 1). The significant probes from these models also demonstrated significant autocorrelation, an alternative statistical test for repeating patterns, in 160 (uncorrected) and 121 (corrected) probes (Table 1). Of these probes, 75 (approximately 50% of the significant seasonal probes) were shared between the uncorrected and corrected datasets.

**Table 1:** Significant probes for cosinor regression.

**Dataset**	**Significant probes**	**Mean *R*^2^ for significant probes**	**Significant autocorrelation**
Uncorrected	169	0.59	160
Corrected	135	0.59	121

The mean variance of gene expression explained by seasonal variation for probe significant at the Bonferroni corrected thresholds.

**Fig 2:**
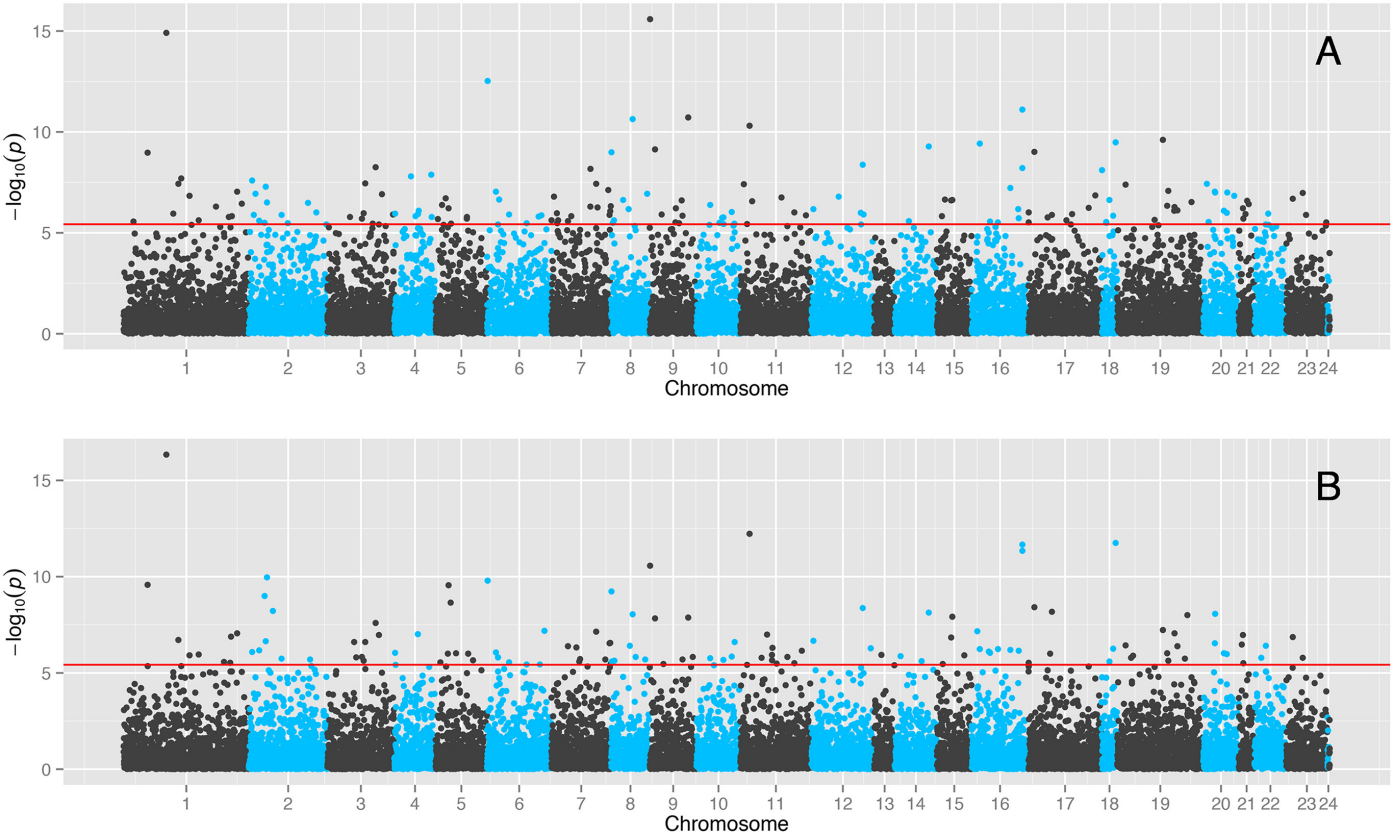
Manhattan plot of the cosinor seasonal analysis. The −*log*10(*p*) of each cosinor regression model is plotted against the chromosomal location of each probe. Bonferroni correction significance line is added. A) Not corrected for cell count B) Corrected for cell count. Includes autosomal chromosomes 1–22, X(23), Y(24) and Mitochondrial(25).

The probes that were significantly associated with season, were located throughout the genome (Fig 2), indicating a diverse range of probes affected by seasonality. The mean proportion of phenotypic variation in expression levels explained by the seasonal effect was 0.13 (uncorrected) 0.12 (corrected) (S1 Fig). The variance explained by the models (*R* ^2^) values for probes with significant levels of association were much higher with both corrected and uncorrected datasets having and *R* ^2^ of 0.59 for (Table 1).

### Cell count seasonality

**Fig 3:**
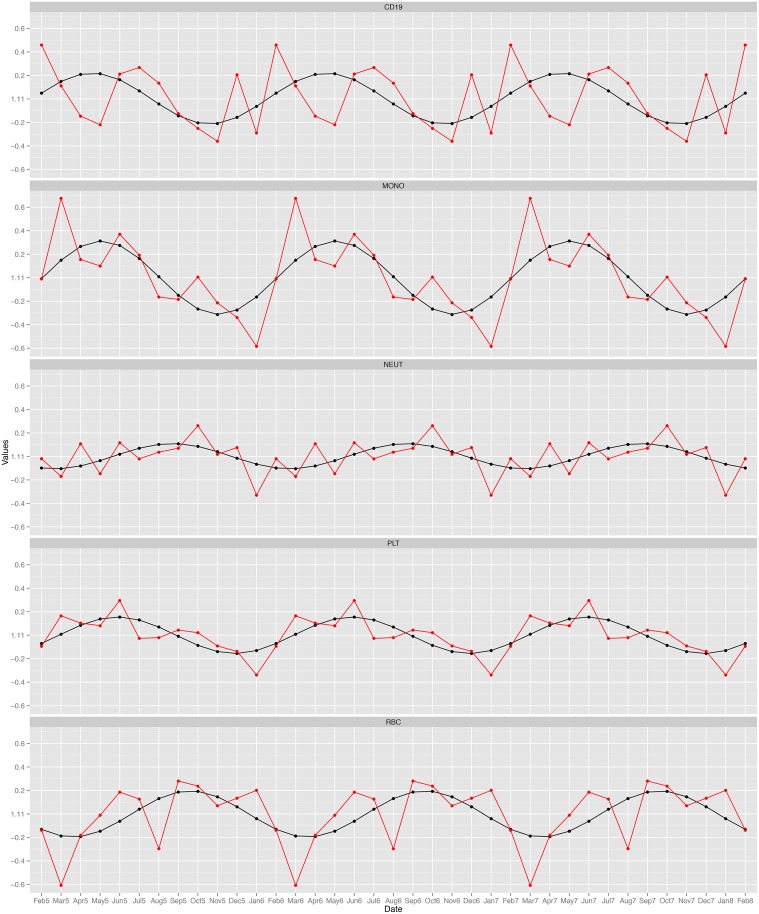
Seasonal variation in cell count. The seasonal variation of five cells that demonstrate significant seasonal variation. The black lines represent the fitted values in a cosinor regression. The red lines represent the actual cell values. From these figures it is evident that the cells follow complex repeating patterns of peaks and troughs throughout the year. However, it can be observed that they show a consistent seasonal trend following one clear peak and trough per year. These values were collected over a three year period and are plotted in sequential order. The year of collection is labeled in the axis as a number (5–8) after the month. This corresponds to the years 2005, 2006, 2007 and 2008 respectively.

Cell counts for 10 blood cell types representing distinct subgrouping in erythrocytes, platelets, granulocytes, monocular cells and lymphocytes were selected for association to seasonal variation using cosinor regression with a 12-month repeating cycle. Five cell types demonstrated a significant association with season: Erythrocytes (*p* = 1.78*e* ^−3^, *R* ^2^ = 0.3), Platelets (*p* = 5.46*e* ^−6^, *R* ^2^ = 0.51), Neutrophils (*p* = 4.41*e* ^−3^, *R* ^2^ = 0.27), Monocytes (*p* = 1.58*e* ^−5^, *R* ^2^ = 0.48) and CD19 cells (*p* = 4.89*e* ^−6^, *R* ^2^ = 0.51). The *R* ^2^ value denotes how much variance in the cell counts the linear model explains. The fitted 12-month seasonal cycle explained between 30–51% of variance in the cell counts. The change in cell count levels throughout the year demonstrates differing seasonal highs and lows (Fig 3). The CD19 cells, Monocytes and Platelets share a similar seasonal cycle that peaks in autumn and drops in spring. Red blood cells and Neutrophils demonstrate a slightly shifted pattern peaking in late winter/early spring and dropping in summer. These seasonal patterns have been reported before for platelets [49] and red blood cells [50].

### Environmental variables

The cosinor regression models a regular cyclic wave that represents natural seasonal variation. For 12 measured environmental conditions ranging from temperature to UV exposure, there was a significant association with a 12 month repeating cosine curve (S2 Fig) with tau-rank correlation coefficient of *τ* ∼ 1 for temperature (ground, maximum and minimum), *τ* ∼ 0.7 for UV, clear days, cloudy days, evaporation, rainy days, rainfall and solar exposure and *τ* ∼ 0.16 for wind speed and hours of sunshine (S2 Fig). The 12-month cycle therefore is a good seasonal surrogate variable that represents various seasonal environmental conditions, in particular temperature, in Brisbane (S2 Fig).

### Enrichment analysis

We next sought to identify a shared biological function of genes exhibiting significant levels of cyclic seasonal variation by performing an enrichment analysis using the Database for Annotation, Visualization and Integrated Discovery (DAVID) [44].

DAVID Functional annotation of the significant seasonal probes for the uncorrected dataset showed enrichment for several Kyoto Encyclopaedia of Genes and Genomes (KEGG) pathways and GO terms related to immune function. This included a number of autoimmune disorders (autoimmune thyroid disease (*p* = 2.3*e* ^−3^), type 1 diabetes (*p* = 4.77*e* ^−4^)), chronic inflammatory diseases (asthma (*p* = 2.1*e* ^−4^)), antigen process and presentation (*p* = 1.97*e* ^−3^) (S3 Fig) cellular activation, differentiation and development (cluster enrichment score 1.8) as well as MHC class 2 immune response (cluster enrichment score 2.28) (Table 2). There was also enrichment for protein production and modification including translation, post-translation modifications and localizations (cluster enrichment score 1.4). Cellular components involved in protein production, the endoplasmic reticulum (*p* = 1.24*e* ^−3^) and the Golgi apparatus membrane (cluster enrichment score 1.44) were also enriched (Table 2). The significant seasonal probes for the corrected dataset, however only showed enrichment for DNA repair and binding (Table 2), a pathway that was also identified for the uncorrected seasonal probes.

**Table 2:** Biological enrichment for the 12 month seasonal cycle.

**Seasonal cycle**	**General process**	**Terms**	**Significance**
Corrected	DNA	DNA repair	CER 1.68
		DNA binding	CER 1.5
Uncorrected	Protein production and modification	Acetylation	*p* = 3.21*e* ^−5^
		Ubiquitin	CER 1.64
		Ribosome biogenesis	CER 1.48
		Protein localization	CER 1.46
		Translation	CER 1.44
		Peptidase activity	CER 1.42
Uncorrected	Cellular component	Endoplasmic reticulum	*p* = 1.24*e* ^−3^
		Golgi apparatus membrane	CER 1.44
	Immune response	Allograph rejection	*p* = 4.19*e*−4
		Asthma	*p* = 2.1*e*−4
		Intestinal immune network for IgA production	*p* = 4.29*e*−4
		Type 1 diabetes mellitus	*p* = 4.77*e*−4
		Autoimmune thyroid disease	*p* = 2.3*e*−3
		Antigen processing and presentation	*p* = 1.97*e*−3
		Lymphocytes differentiation	CER 1.6
		Antigen processing and presentation	CER 1.5
		Immune cell activation differentiation and development	CER 1.8
		MHC class two immune response pathway	CER 2.28
Uncorrected	DNA	DNA binding	CER 1.75
		Nucleotide metabolism	CER 1.67
Uncorrected	Cellular function	Apoptosis	CER 1.93

CER = cluster enrichment score

### Cell-specific mRNA markers

As the expression levels were measured in whole blood, which is composed of many cell types, we attempted to determine whether a specific cell type drove the seasonal expression patterns. Using the userListEnrichment() function incorporated in the WGCNA R package, we tested 11 lists of different blood cell markers for enrichment. This analysis revealed that the non-corrected seasonal probes were significantly enriched for lymphocyte markers (Table 3) after Bonferroni correction (0.05/11 test sets). After correction for cell count, no association to any cell markers were observed, indicating that cellular markers can reflect the cell counts present for the individuals. This demonstrates that fluctuations in cell count can be observed within the transcriptome through the presence of specific cellular markers and also through the enrichment of seasonal genes involved in immune function.

**Table 3:** Gene list enrichment analysis for blood cells.

**User Defined Categories**	**Type**	**Number of Genes**	**Corrected p-values**	**Genes**
Bcell Blood (composite)	Blood	31	3.52E-06	BANK1, BCL11A, C22ORF13, C4ORF34, CCDC106, CCR6, CD24, CD79A, CD79B, CXXC5, CYBASC3, EIF2AK3, GJB6, GNB5, HLA-DOA, HVCN1, ITPR1, IVD, MEF2C, NOC3L, P2RY10, PACAP, PNOC, SMARCB1, SP100, SPIB, TLR10, TPD52, TTC21A, ZDHHC23, ZNF165
Lymphcytes genesCorrelatedAcrossIndividuals Whitney	Blood	11	3.2e-02	BTG1, CD74, CD79A, CSF1R, HLA-DMB, HLA-DPA1, HLA-DRA, HLA-DRB4, MS4A1, SPIB, TCL1A

Enrichment for blood cell signature was found using the userListEnrichment function in the WGCNA R package.

## Discussion

There are seasonal differences in the prevalence and severity of conditions such as psychiatric disorders [27] [51] [30] [29], inflammatory [25] and cardiovascular diseases [52] [26]. The effect of seasonal variability has also been recorded for several molecular phenotypes such as homovanillic acid [53], serotonin [54] [55], monoamine neurotransmitters [56], 25-hydroxyvitamin D3 [57] [58] [59] [60] and N-3 poly-unsaturated fatty acid [61]. Gene expression provides an intermediate phenotype between the genome and higher order phenotypes such as metabolites and can provide clues as to the underlying biological functions that are being altered in response to seasonal changes.

We investigated whole blood gene expression for seasonal variability in a cohort of 606 individuals. Using cosinor regression models we were able to identify 135 probes (1% of all probes tested) that showed significant seasonal variation after being corrected for blood cell composition. Significant autocorrelation, an alternative statistical technique, was also identified for 90% of these probes further confirming the presence of repeating cyclic trends in expression levels of numerous transcripts. In order to examine how this impacts seasonal gene expression, we examined the expression levels with and without corrections for cell counts.

Transcripts showing seasonal variation in the uncorrected data were enriched for immune pathways and protein production. The enriched KEGG immune pathways included (Table 2) allograph rejection, antigen processing and presentation (S3 Fig), lymphocyte differentiation, immune cell activation, differentiation and development, MHC class two immune response and autoimmune diseases; type 1 diabetes, autoimmune thyroid disease and asthma. Asthma is a chromic inflammatory disease condition, which has been observed to exhibit seasonal variability [25], potentially through the cellular mechanisms identified here. Other cellular function, such as protein modification and apoptosis, were found in the uncorrected gene expression dataset (Table 2). There were 74 seasonally associated genes shared between the corrected and uncorrected datasets and these were enriched with terms for DNA binding. This suggests that transcripts encoding genes involved with DNA binding experience significant seasonal variation, independent of seasonal fluctuations in cell count.

A previous study by De Jong et al. [20] identified three modules, comprising 5,062 probes (mapping to 1,458 unique genes), that were associated with cyclic seasonal patterns. However, these probes were primarily driven by changes in red blood cells and platelets [20]. Here, we did not identify cell type specific gene signatures for red blood cells, but instead identified enrichment for leukocytes markers. This difference could be attributed to the single-gene approach we employed and that we only shared 2,406 genes (18% of our dataset) with the De Jong et al. analysis [20].

We demonstrate in this study that the 12-month cosinor regression has perfect correlation (*τ* ∼ 1) with temperature, a high correlation (*τ* ∼ 0.7) with UV index, number of clear, cloudy and rainy days, evaporation, rainfall and solar exposure, and a low correlation (*τ* ∼ 0.16) for wind speed and hours of sunshine. This relationship suggests that temperature could be a major factor driving the seasonal variation in gene expression levels identified with the 12-month cosinor regression model.

A limitation of this study is that each time point represents the mean expression levels of a group of samples collected during the same time period. Therefore, estimates of effects represent population variation, rather than intra-individual variation. To more accurately assess the impact of seasonal environmental factors of gene expression, repeat measures should be collected for samples throughout the year.

## Conclusion

Our results demonstrate the effect of seasonality on cell count and gene expression levels. We observe that the cellular composition of erythrocytes, platelets and leukocytes varies throughout the year, following seasonal patters. This trend was also evident in gene expression levels, with significant seasonal changes in the expression of genes involved in immune function and protein translation. However, we are unable to determine the direct route of causation for these transcriptional changes. DNA binding however, a key component of transcription control, demonstrated significant seasonal variation independent of cell counts, indicating that gene expression could be pervasively influenced by seasonality in a regulatory manner. Collectively, our results show that seasonality is an important environmental regulator of physiological processes, which can be identified through transcriptional variation.

## Supporting Information

S1 Fig Histograms showing the distribution of the variance explained by the model (*R* ^2^). Blue denotes probes with statistically levels of association between gene expression levels and cyclic variation A) Not corrected for cell count B) Corrected for cell count.
(TIFF)

S2 Fig Standardized monthly values for 12 weather conditions. Measured weather variables that exhibit seasonal variation in Brisbane (black dots and connecting lines). The red dots represent the cosine curve with a 12-month repeating cycle.
(TIF)

S3 Fig KEGG enriched pathway for Antigen Processing and Presentation. Significant seasonal genes in our study are highlighted with red stars.
(TIF)

S1 Table A table containing all seasonal data for BSGS cohort samples. Sample IDs match those of the expression data deposed in GEO GSE33321
(TXT)
