# Correction: Seasonal Effects on Gene Expression

**DOI:** 10.1371/journal.pone.0316470

**Published:** 2024-12-20

**Authors:** Anita Goldinger, Konstantin Shakhbazov, Anjali K. Henders, Allan F. McRae, Grant W. Montgomery, Joseph E. Powell

The accession number appearing in the Data Availability statement is incorrect. The correct Data Availability statement is: All gene expression and corresponding batch, age and sex covariate data, cell count data and family-corrected gene expression data have been deposited in GEO (accession number: GSE53195). All seasonal data was obtained from the publicly accessible Australian Bureau of Meteorology (http://www.bom.gov.au/) and the Australian Radiation Protection and Nuclear Safety Agency (http://www.arpansa.gov.au/) and are available in the supplementary information of the paper.
