# Supplementary figures and images for: Seasonal Effects on Gene Expression
Source: PLoS One. 2015 May 29;10(5):e0126995. doi: 10.1371/journal.pone.0126995 (PMC4449160; doi:10.1371/journal.pone.0126995)

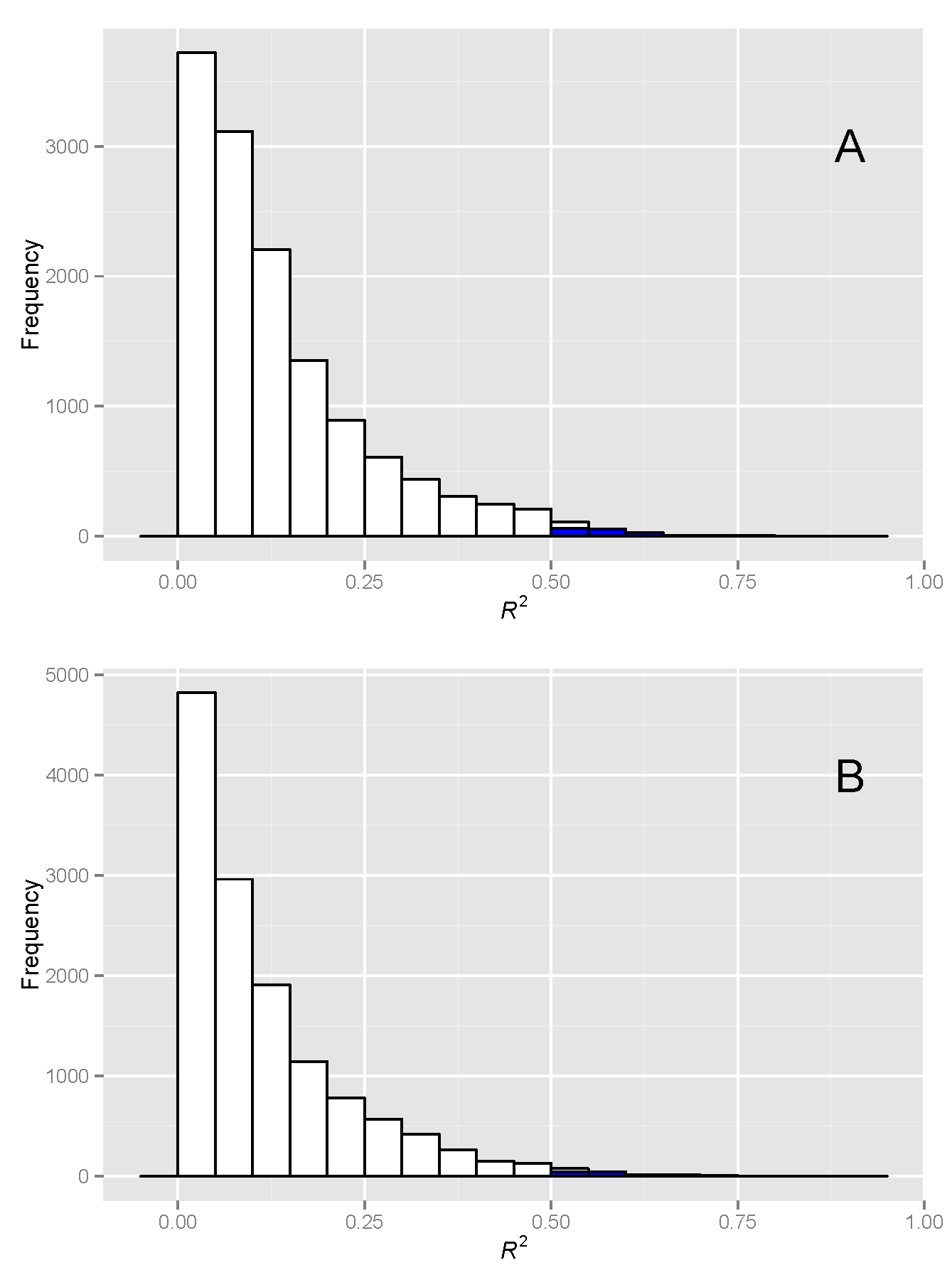

Supplement: S1 Fig — Blue denotes probes with statistically levels of association between gene expression levels and cyclic variation A) Not corrected for cell count B) Corrected for cell count. (TIFF) [file pone.0126995.s001.tiff]

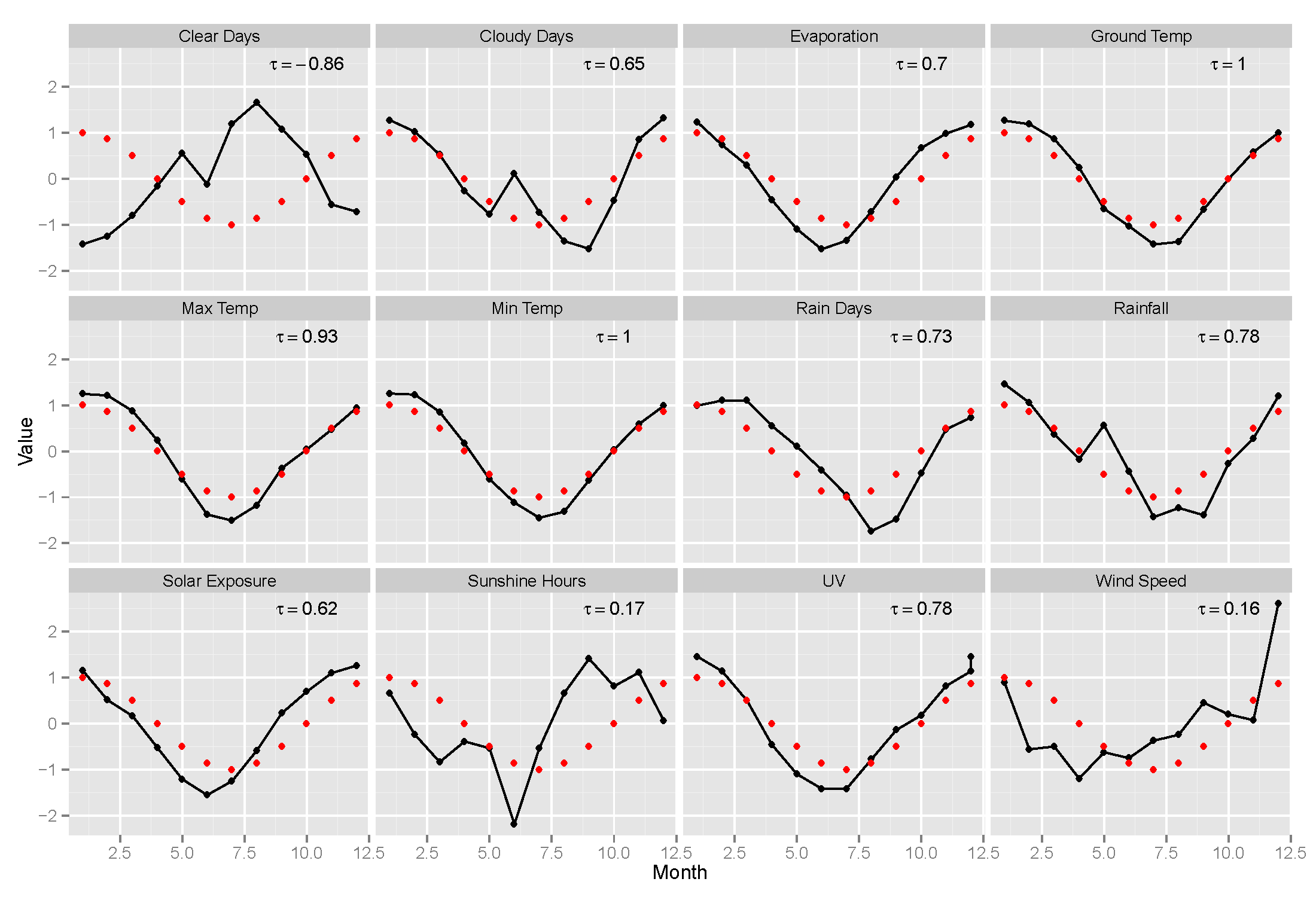

Supplement: S2 Fig — Measured weather variables that exhibit seasonal variation in Brisbane (black dots and connecting lines). The red dots represent the cosine curve with a 12-month repeating cycle. (TIF) [file pone.0126995.s002.tif]

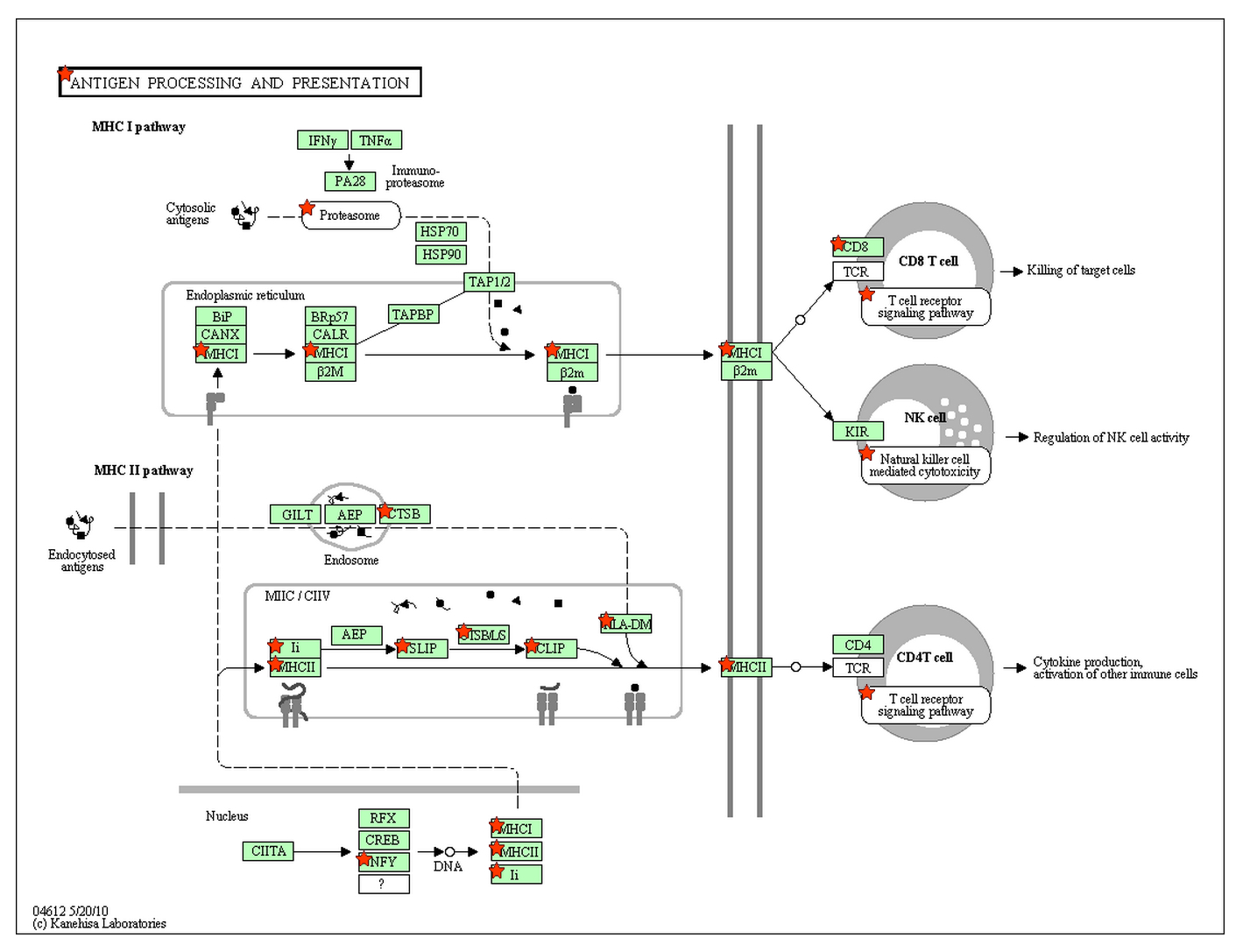

Supplement: S3 Fig — Significant seasonal genes in our study are highlighted with red stars. (TIF) [file pone.0126995.s003.tif]
